# Overexpression of HIF-1α indicates a poor prognosis in tongue carcinoma and may be associated with tumour metastasis

**DOI:** 10.3892/ol.2013.1185

**Published:** 2013-02-07

**Authors:** YANG ZHENG, YANHONG NI, XIAOFENG HUANG, ZHIYONG WANG, WEI HAN

**Affiliations:** 1 Department of Oral and Maxillofacial Surgery, Stomatological Hospital Affiliated Medical School, Nanjing University, Nanjing 210008, P.R. China; 2 Central Laboratory of Stomatology, Stomatological Hospital Affiliated Medical School, Nanjing University, Nanjing 210008, P.R. China; 3 Department of Pathology, Stomatological Hospital Affiliated Medical School, Nanjing University, Nanjing 210008, P.R. China

**Keywords:** hypoxia-inducible factor 1, tongue carcinoma, prognosis

## Abstract

Expression of the transcription factor hypoxiainducible factor 1 (HIF-1) plays a key role in cellular adaptation to hypoxia, particularly in relation to tumour angiogenesis. Expression of the HIF-1α subunit is responsive to changes in oxygen levels. Overexpression of HIF-1α has been reported to be associated with a poor prognosis in a variety of malignant tumours. The objective of this study was to investigate whether the expression of HIF-1α in tongue carcinoma was associated with established clinicopathological features. Tumour specimens from 120 patients with histologically-proven, surgically-treated tongue carcinoma were examined by immunohistochemical staining for expression of HIF-1α. The mRNA levels of HIF-1α were measured in 45 fresh, paired samples of tongue carcinoma and corresponding adjacent normal tissues using quantitative RT-PCR (qRT-PCR). HIF-1α was found to be frequently overexpressed in tumours in a hypoxia-independent manner. The expression of HIF-1α correlated with the five-year survival rate (P<0.01) and disease-free period (P<0.01). Increased expression of HIF-1α correlated significantly with clinical stage (P=0.002) and lymph node metastasis (P=0.034). Compared with paired normal tissues, HIF-1α mRNA levels were significantly increased in carcinoma of the tongue. A positive correlation was observed between HIF-1α mRNA levels and pathological differentiation grade. A significant difference in the levels of HIF-1α expression was detected between groups of patients with lymph node metastases and patients with no metastases. These results indicate that overexpression of HIF-1α may be an indicator of poor prognosis in carcinoma of the tongue. The expression of HIF-1α may be associated with lymph node metastasis.

## Introduction

Hypoxia, a frequent characteristic of solid tumour growth in head and neck cancer and other cancers, stimulates a cascade of molecular pathways resulting in angiogenesis, glycolysis and changes in the cell cycle. The dimeric transcription factor hypoxia-inducible factor (HIF-1) is a key regulator of the cellular response to hypoxia (1,2). HIF-1 functions as a master regulator of oxygen homeostasis and undergoes conformational changes in response to different oxygen concentrations (3). HIF-1 consists of two subunits, α and β, which are both helix-loop-helix transcription factors. The HIF-1α subunit mediates HIF-1 function as a transcription factor in response to cellular hypoxia (4). Alteration and overexpression of HIF-1α has been detected in a variety of solid tumours, including breast, lung, ovarian, and head and neck cancers, with varying (diffuse and perinecrotic) staining patterns. The expression of HIF-1α was determined to be of prognostic relevance in different tumours (5–8). However, based on a review of the literature (9), the prognostic relevance of HIF-1α in tumours derived from squamous epithelium remains controversial. In clinical specimens, elevated HIF-1α expression correlates with poor outcome in colorectal, pancreatic, breast, cervical, endome-trial, ovarian, bladder, gastric, and head and neck carcinomas. There is a growing body of evidence suggesting that HIF-1α is involved in the progression of oral cancers (10–13). In clinical studies, elevated expression of HIF-1α was found to correlate with lymph node involvement, tumour-node-metastasis (TNM) classification, poor survival and resistance to chemo- and radiotherapy in patients with oral squamous cell carcinomas (12,14,15). Contradictory results relating to the role of HIF-1α in oral carcinomas have also been reported, however (16). Carcinoma of the tongue represents a significant proportion of oral cancers. The tongue has a rich blood supply, so it was of interest to examine whether hypoxic conditions existed in this tissue. The aim of this study was, therefore, to verify the role of HIF-1α expression in carcinoma of the tongue, to evaluate its correlation with clinicopathological features and to determine its value as a prognostic marker.

## Materials and methods

### Patients and specimens

The cohort was assembled from patients who were histologically diagnosed with carcinoma of the tongue and who underwent radical surgery at the Department of Oral and Maxillofacial Surgery, Stomatological Hospital Affiliated Medical School, Nanjing University, between 2000 and 2008. Exclusion criteria included recurrence at presentation, pre-operative radiotherapy, chemotherapy or hormone therapy, residual tumour at the surgical margin or incomplete medical records. All medical records were reviewed retrospectively, according to the inclusion and exclusion criteria. Table I summarises the characteristics of patients (n=120) with carcinoma of the tongue that were recruited to this study. The median age of the patients at the time of diagnosis was 57 years (range 33–81 years). The formalin-fixed, paraffin-embedded specimens from these patients were used for immunohistochemical analysis. The follow-up period was calculated from the date of surgery to the date of death, loss of follow-up, or the 60th month, whichever came first (median follow-up period, 47 months).

The study was approved by the Ethics Committee of Stomatological Hospital Affiliated Medical School, Nanjing University, Nanjing, China. Written informed consent was obtained from the patients.

### Immunohistochemical staining

Sections (4 μm) were deparaffinised in xylene and rehydrated. Antigen retrieval was performed using the heat-induced epitope retrieval method. Slides were boiled in antigen retrieval buffer (0.001 mol/l EDTA solution, adjusted to pH 8.0) in a pressure cooker until full pressure was reached, and maintained for another 90 sec. After the slides were cooled to room temperature, they were incubated with a mouse monoclonal antibody to human HIF-1α (1:1200) for 4 h at room temperature and at 4°C overnight. Instead of the primary antibody, the negative control was incubated with phosphate-buffered saline (PBS; pH 7.4). The slides were washed twice with PBS for 5 min and tissues were incubated with a specific biotinylated secondary antibody, the streptavidin-biotin-peroxidase complex system (Novolink Max Polymer Detection System, Novocastra, Newcastle-upon-Tyne, UK) at 37°C in a thermostatically-controlled container for 40 min. The slides were washed twice for 5 min with PBS and the colour was developed with 3,3′-diaminobenzidine. The slides were counterstained with Mayer’s haematoxylin, washed, dehydrated and cleared. Coverslips were sealed with neutral balsam. The percentage of positive cells was estimated using an image analysis system.

The level of expression of HIF-1α was determined independently by three pathologists. Each pathologist determined the percentage of positive cell nuclei in each field. Tissues were scored according to the percentage of positive immunostaining (P) as follows: 0 (<1%), 1 (1–5%), 2 (5–10%), and 3 (>10%).

### RNA preparation and real-time quantitative RT-PCR

Total RNA was extracted from 45 fresh, paired samples of tongue carcinoma and corresponding adjacent normal tissues using TRIzol reagent (Invitrogen, Life Technologies, Gaithersburg, MD, USA) following the manufacturer’s protocol. Real-time quantitative RT-PCR (qRT-PCR) was performed using the Thermal Cycler Dice™ Real-Time System TP800 (Takara) according to the standard protocol of the SYBR^®^ Premix Ex Taq™ Perfect Real-Time system (Takara). Primers for HIF-1α were as follows: sense 5′-TGTGAACCCATTCCTCAC CCATCA-3′, antisense 5′-CAGTTTCTGTGTCGTTGCTGC CAA-3′ and for β-actin: sense 5′-TCACCCACACTGTGCC CATCTACGA-3′ and antisense 5′-CAGCGGAACCGCTCA TTGCCAATGG-3′. Thermal cycling conditions were 95°C for 1 min, followed by 40 cycles of 95°C for 15 sec and 60°C for 1 min. The level of expression of HIF-1α in the tumour and adjacent normal samples was quantified by measuring the fractional cycle number at which the level of expression reached a fixed threshold (Ct) and this was directly related to the amount of product. The housekeeping gene, β-actin, was used as an internal control to quantify the products of HIF-1α. The relative quantification was given by the Ct values, determined for triplicate reactions for tongue carcinoma and adjacent normal samples for HIF-1α and β-actin. The average of triplicate Ct values for HIF-1α was determined and the average β-actin Ct value was subtracted from the mean HIF-1α Ct value, to obtain ΔCt (ΔCt = Ct(target gene in carcinoma of tongue/adjacent normal sample) - Ct(β-actin gene in carcinoma of tongue/adjacent normal sample). Relative expression level was determined as 2*^–^*^ΔΔC^*^t^*, where ΔΔCt=ΔCt (carcinoma sample)-ΔCt (adjacent normal sample). Therefore, 2*^–^*^ΔΔCt^ indicates the fold change in HIF-1α expression in carcinoma samples relative to adjacent normal samples.

### Statistical analysis

SPSS 18.0 was used for statistical analysis. TNM stage, histological differentiation status and expression of HIF-1α were correlated with the duration of progression-free and overall survival. Progression-free survival and overall survival were calculated from the date of surgery to the date of histologically-proven recurrent or metastatic carcinoma, or disease-related mortality, respectively. Patients who succumbed to intercurrent diseases were censored at the date of mortality. Patients lost to follow-up were censored at the date of the last examination. The progression-free survival curves and overall survival curves were constructed according to Kaplan and Meier. The log-rank test was used to assess differences between groups and multivariate survival analysis was performed with multivariate Cox regression analysis. Correlations between clinicopathological features and expression of HIF-1α were evaluated using χ^2^ test. The correlation between relative mRNA expression and pathological characteristics was analysed using non-parametric tests. P<0.05 was considered to indicate a statistically significant result.

## Results

### HIF-1α expression and clinical outcome

Moderate to strong staining was observed in 81.8% (54/66) of advanced-stage tumours and 75% (36/48) of tumours with lymph node metastasis. In contrast, weak to moderate staining was observed in 38.9% (21/54) of early-stage tumours and 54.2% (39/72) of tumours with no lymph node metastasis. The correlation between HIF-1α expression and lymph node metastasis or clinical stage was indicated by Spearman correlative analysis (Table II, P= 0.034 and P=0.002, respectively). HIF-1α expression did not correlate with gender, age, histological differentiation or location of primary sites.

**Figure 1:**
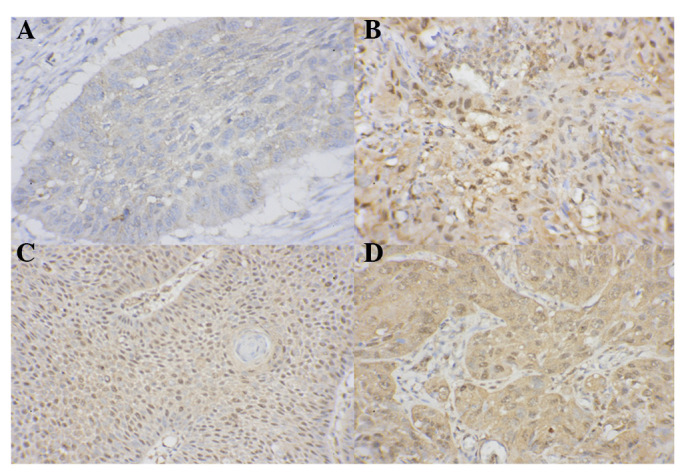
Representative immunohistochemical images of hypoxia-inducible factor-1α (HIF-1α). (A) Expression of HIF-1α in negative control (magnification, ×100). (B) Expression of HIF-1α in well-differentiated group (magnification, ×100). (C) Expression of HIF-1α in moderately differentiated group (magnification, ×40). (D) Expression of HIF-1α in poorly differentiated group (magnification, ×100).

**Figure 2:**
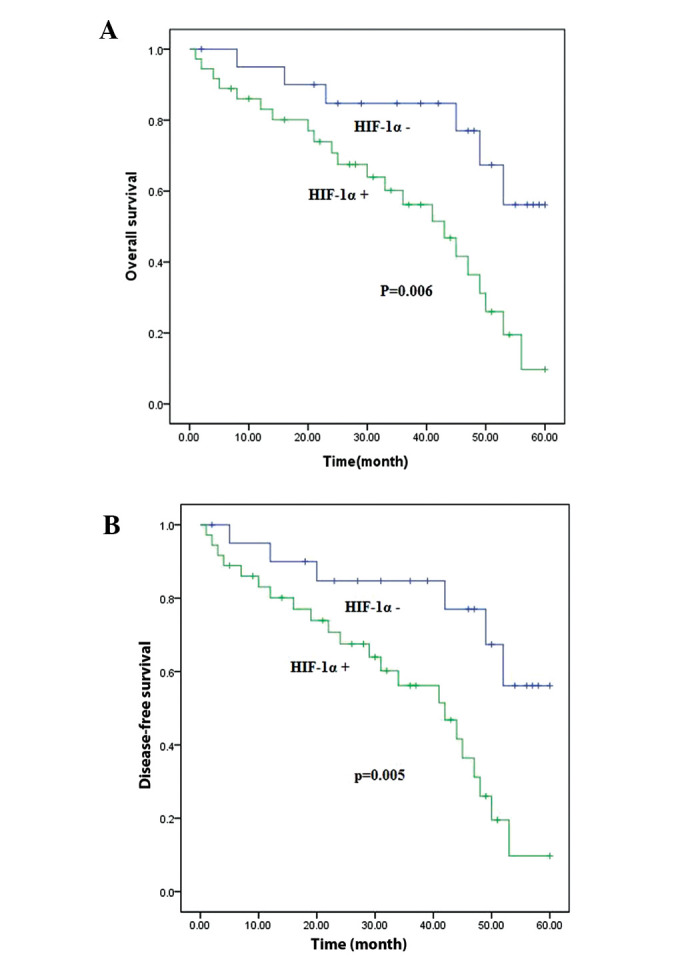
Expression of hypoxia-inducible factor-1α (HIF-1α) in carcinoma of the tongue specimens and their correlations with (A) overall survival and (B) disease-free survival.

**Figure 3:**
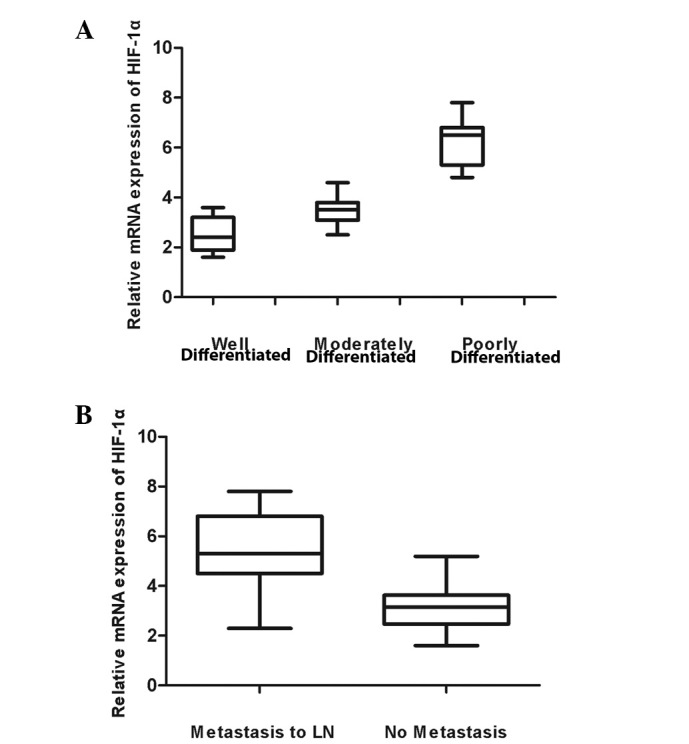
mRNA expression of hypoxia-inducible factor 1α (HIF-1α) (A) among different pathological grades and (B) in groups of patients with lymph node metastasis and no metastasis.

Representative immunohistochemical images are shown in Fig. 1. Nuclear staining of HIF-1α was detected in 75% (90/120) tongue carcinoma specimens. The Kaplan-Meier survival analysis revealed a significantly worse overall survival (P<0.001) and disease-free survival (P<0.001) for patients with nuclear staining of HIF-1α compared with those whose tumours were negative for HIF-1α staining (Fig. 2). The correlations between the relative mRNA expression of HIF-1α and pathological characteristics of the tumour sample, such as TNM stage, clinical stage, pathological differentiation grade and smoking and drinking were analysed (Table II). A positive correlation was found between the mRNA level of HIF-1α and the pathological differentiation grade of tongue carcinomas. Furthermore, the expression of HIF-1α was significantly different between groups of patients with lymph node metastasis and no metastasis (P<0.05; Fig. 3).

## Discussion

The correlation between HIF-1α expression and oral cancers has been widely reported. The actual role of HIF-1α in tongue carcinoma progression, however, remains unclear. The tongue is supplied by abundant blood vessels. Regions of hypoxia are likely to be very limited in this tissue, therefore. The aim of this study was to examine the role of HIF-1α signalling in tongue carcinoma progression. The results of this study reveal that, although the blood supply in the tongue is abundant, there was evidence of a hypoxic state in the tumours, possibly due to their rapid growth. This suggests that HIF-1α may play an important role in the progression of tongue carcinoma.

Several previous studies have provided evidence that HIF-1α plays different roles in different types of tumours. Higher levels of HIF-1α expression in this study correlated strongly with lymph node metastasis, which indicates that HIF-1α is involved in tongue cancer metastasis. Determining the level of expression of HIF-1α may, therefore, provide valuable information to inform the choice of treatment or to assess prognosis. In agreement with previous reports (12,17,18), this study showed that HIF-1α expression correlated with overall survival, and disease-free survival, of patients with tongue carcinomas. It was suggested that rapid tumour growth resulted in a large tumour size and areas of hypoxia, which induces the accumulation of HIF-1α (12). Once stabilised, HIF-1α transactivates a set of downstream genes that, in turn, facilitate tumour growth, angiogenesis and metastasis. In this regard, the correlation between HIF-1α expression and patient survival is easily understood. It has been reported that targeting HIF-1α signalling in tumour cells significantly inhibits tumour growth in mouse xenografts (19–23). Raval *et al*(24) found that HIF-1α expression was associated with improved disease-free survival in surgically-resected head and neck squamous cell carcinoma, however. Recent studies have revealed a tumour suppressive role of HIF-1α, probably due to HIF-1α-induced apoptosis or transactivation of specific genes that are targets for negative selection in human cancers (25). In light of these discrepancies among different tumours there may be tissue-specific differences in response to HIF-1α regulation. In this study, increased expression of HIF-1α was observed in more advanced stages of tongue cancer, which indicates the critical role of HIF-1α in tongue carcinogenesis. More importantly, the expression of HIF-1α correlated closely with lymph node metastasis and could be a reliable marker to predict the prognosis of tongue cancer.

Overexpression of HIF-1α could be an indicator of poor prognosis in carcinoma of the tongue. HIF-1α overexpression correlated with clinical stage and lymph node metastasis in patients with carcinoma of the tongue.

## Figures and Tables

**Table I:** Summary of demographic and clinical parameters.

Variable	No.	%
Age (years, mean ± SD)	57±10.1	
Gender		
Female	54	45.0
Male	66	55.0
Alcohol and tobacco		
Yes	36	30.0
No	84	70.0
Lymph node metastasis		
Yes	48	40.0
No	72	60.0
Clinical stage		
Early (I/II)	54	45.0
Advanced (III/IV)	66	55.0
Pathological grade		
I	45	37.5
II	70	58.3
III	5	4.2

**Table II:** Correlation between clinical parameters of patients with carcinoma of the tongue and hypoxia-inducible factor-1α (HIF-1α) expression levels.

Categorical variables	HIF-1α (nuclear)		
	Negative	Positive	P-value
Gender			
Male	20	46	
Female	25	29	0.072
Alcohol and tobacco			
Yes	14	22	
No	31	53	0.837
Lymph node metastasis			
Yes	25	27	
No	20	48	0.036^a^
Clinical stage (T stage)			
T1/T2	27	27	
T3/T4	18	49	0.009^a^
Tumour grade (differentiation)			
Poor	17	21	
Moderate	24	48	
Well	4	6	0.16

a P<0.05.
